# Solitary accessory and papillary muscle hypertrophy manifested as dynamic mid-wall obstruction and symptomatic heart failure: diagnostic feasibility by multi-modality imaging

**DOI:** 10.1186/1471-2261-14-34

**Published:** 2014-03-10

**Authors:** Kuo-Tzu Sung, Chun-Ho Yun, Charles Jia-Yin Hou, Chung-Lieh Hung

**Affiliations:** 1 Division of Cardiology, Department of Internal Medicine, Mackay Memorial Hospital, Taipei, Taiwan; 2 Department of Radiology, Mackay Memorial Hospital, Taipei, Taiwan; 3 Department of Medicine, Mackay Medical College, and Mackay Medicine, Nursing and Management College, Taipei, Taiwan; 4 Cardiovascular Medicine, Mackay Memorial Hospital, No. 92, Section 2, Chung Shan North Road, Taipei, Taiwan

**Keywords:** Solitary papillary muscle hypertrophy, LVOT obstruction, Hypertrophic cardiomyopathy

## Abstract

**Background:**

Solitary papillary muscle (PM) hypertrophy is an unique type of hypertrophic cardiomyopathy (HCM), which is characterized by predominant papillary muscle hypertrophy sparing the rest of other left ventricular segments. It has recently drawn our attention about the mechanism of left ventricular mid-cavity obstruction and the influence of pressure gradient in the left ventricular outflow tract (LVOT), thus carries clinical importance.

**Case presentation:**

We reported a symptomatic, 83-year-old woman who presented with dynamic, high resting left ventricle (LV) mid-wall gradient without obvious septal hypertrophy or systolic anterior motion (SAM). Subsequent real-time (RT) three-dimensional echocardiography (3DE) and cardiac magnetic resonance imaging (MRI) demonstrated large, hypertrophic accessory papillary muscles squeezing mid-cavity of left ventricle producing dynamic pressure gradient during systole in the absence of left ventricular wall anomalies.

**Conclusion:**

We proposed that combined use of echocardiography particularly RT-3DE and cardiac magnetic resonance imaging (MRI) can accurately identify this specific type of hypertrophic cardiomyopathy without remarkable traditional features.

## Background

Hypertrophic obstructive cardiomyopathy (HOCM) refers to those subjects with significant dynamic left ventricular outflow tract (LVOT) obstruction due to mechanical causes, with most presents with asymmetrical septal hypertrophy resulting in dynamic systolic anterior motion (SAM) of mitral leaflets [1].

Solitary papillary muscle (PM) hypertrophy, a peculiar form of HCM manifested as predominant PM hypertrophy sparing the rest of other LV segments, has recently gained much attention owing to its mechanical consequences on left ventricular outflow tract (LVOT) pressure gradient formation [2,3].

## Case presentation

**Figure 1:**
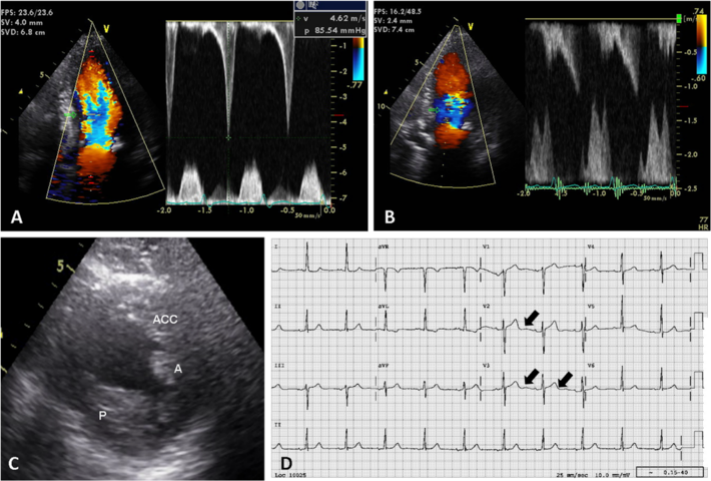
**2D Echocardiography and complete ECG. (A)** Continuous wave Doppler tracing of the LV mid-wall showed the peak pressure gradient was 85.5 mmHg at rest. **(B)** Subsequent continuous wave Doppler tracing eight hours later showed normal pressure gradient (estimated to be 5 mmHg). **(C)** In short axis view, apparently hypertrophied anterolateral and posteromedial papillary muscles together with a large, third accessory PM were observed (A: anterolateral papillary muscle, P: posteromedial papillary muscle, ACC: accessory papillary muscle). **(D)** Complete ECG showed prominent U wave in V1-V4.

A 83-year-old woman with history of hypertension and hyperlipidemia. The blood pressure and hyperlipidemia were well-controlled with medication. She was admitted to our hospital with chest pain on effort and exertional shortness of breath. Her vital sign was stable with blood pressure 131/70 mmHg and heart rate 67 beats per minute at initial presentation. A mid-systolic ejection murmur was heard along the left sternal border with chest X-ray showed minor pulmonary congestion. Incidental high resting LV mid-wall pressure gradient (86 mmHg, Figure 1A) was shown by 2-dimensional echocardiography without LV septal wall thickness (8.5 mm). The LV end-diastolic volume was 90 ml, and LV end-systolic volume was 27 ml. Apparently hypertrophied anterolateral (A) and posteromedial (P) papillary muscles together with a large, third accessory PM were observed (Figure 1C). Neither aortic stenosis nor SAM was observed. Complete ECG disclosed obvious U wave (V1-V4) with normal QT interval (416ms, Figure 1D). Blood level of B-natriuretic peptide was obviously elevated (227 pg/ml).

**Figure 2:**
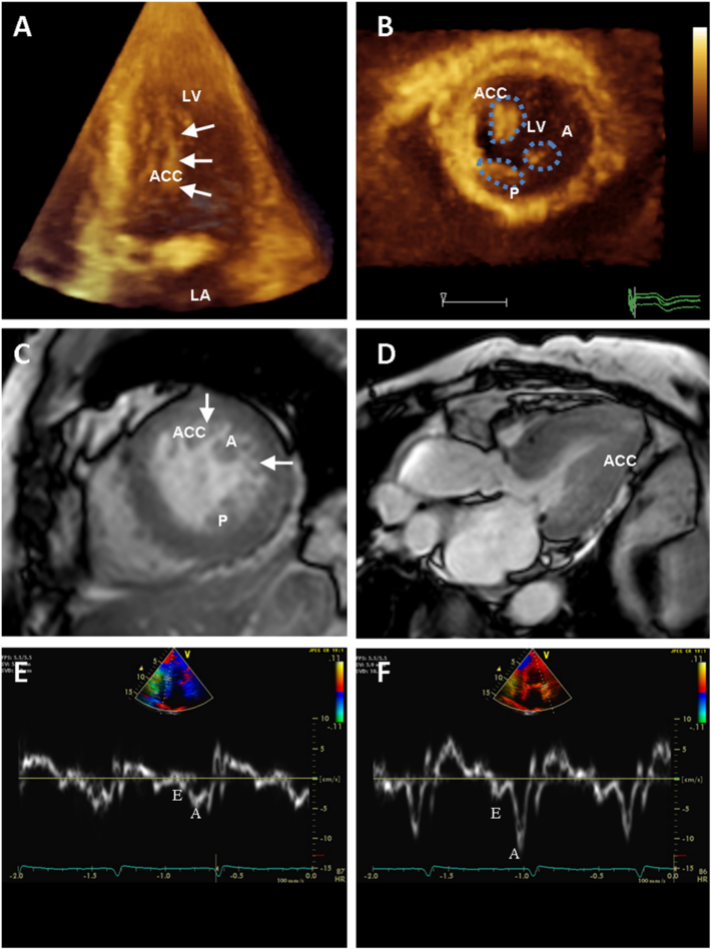
**Real 3D echocardiography, MRI, and tissue doppler imaging. (A)** 2D echocardiogram in the short-axis section of the left ventricle demonstrated uncommonly enlarged papillary muscles (A: anterolateral papillary muscle, P: posteromedial papillary muscle, ACC: accessory papillary muscle). **(B)** Real time 3D Echocardiography demonstrated that LV mid-cavity was almost obliterated during end-systolic phase from five chamber view (Additional file 1, movie 1). A: anterolateral papillary muscle, LA: left atrium, LV: left ventricle. **(C)** Contrast-enhanced MRI from LV short-axis showed two large papillary muscles as well as adjacent abundant muscular trabeculae producing mid-cavity obstruction during systolic phase. **(D)** MRI from long-views showed anterolateral papillary muscle squeezing left ventricular outflow tract against LV septum during systolic phase. High temporal resolution Tissue Doppler Imaging showed markedly diminished diastolic mitral annulus relaxation velocities, 1.8 cm/sec for basoseptal **(E)** and 4.3 cm/sec for basolateral segments **(F)**.

**Figure 3:**
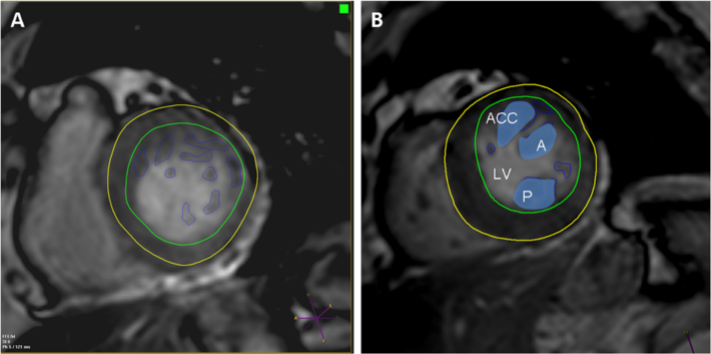
**Cardiac MRI, short axis view. (A)** Unremarkable LV wall abnormality with normal LV mass at end-diastolic phase by MRI; **(B)** Hypertrophy PMs as well as existence of accessory PM (ACC) shown.

Repeated 2D transthoracic echocardiogram during hospitalization revealed no more pressure gradient (plummeted to 5 mmHg) after holding diuretics (Figure 1B). Meanwhile, she was in euvolemic state with blood pressure 128/76 mmHg and heart rate 62 beats per minute. To further clarify the spatial relationships between these hypertrophied PMs and other LV wall segments, real-time 3D echocardiography (RT-3DE) was performed and showed nearly complete LV mid-wall cavity obliteration by the these morphological abnormal PMs during end-systolic phase (Figure 2A-B; Additional files 1, 2 and 3). Subsequent MRI study demonstrated normal LV volume (end-diastolic volume: 71.9 ml), unremarkable LV wall abnormalities, normal global LV mass index (79.9 gm/m^2^) though significantly hypertrophied PMs (total PM mass: 20.2 gm; 13.4 gm/m^2^ indexed to body surface area; Figure 2C-D) without visible perfusion defects or delayed hyperenhancement (DHE). High temporal resolution Tissue Doppler Imaging (>250 frames per second) further demonstrated markedly diminished diastolic mitral annulus relaxation velocities (1.8 cm/sec for basoseptal and 4.3 cm/sec for basolateral segments; Figure 2E-F). When PMs mass were taken into account (up to 16.8% LV mass; Figure 3A-B), the total LV mass may reach the echocardiographic criteria for LV hypertrophy [4] (defined as LV mass index >95 gm/m^2^ in women and > 115 g/m^2^ in men). Beta-blockade was then prescribed and the symptoms improved drastically during the subsequent follow up.

## Conclusions

So far, left ventricular hypertrophy remained the predominant and major phenotypic component of hypertrophied cardiomyopathy [1]. Few cases of dynamic LVOT or mid-cavity obstruction in relation to various morphological papillary muscle anomalies had been reported before, and these may include bifid [5] or octopus papillary muscle [6], accessory [7], single [2], or solitary papillary muscle hypertrophy [8]. Papillary muscle hypertrophy, when defined by at least one of the two papillary muscles is more than 1.1 cm in either vertical or horizontal diameter, had been recently shown to be a phenotypic variant of HCM [8,9]. Not until recently, morphological papillary muscle anomalies without features of phenotypic LV hypertrophy (isolated papillary muscle hypertrophy) had gradually been recognized to be an uncommon HCM variant [8,10]. The clinical features of LV mid-cavity obstruction caused by papillary muscle hypertrophy may vary greatly from asymptomatic to dyspnea, angina, syncope, and even sudden cardiac death [8-10], with prominent U wave and left ventricular hypertrophy by ECG [11,12], which is concordant with our current case findings.

While HCM subjects with concomitant morphological papillary muscle anomalies may present with higher degree of resting LVOT gradient [13], it is not surprising that subjects with increasing number of hypertrophied papillary muscles with unchanged ventricular cavity size may have higher probability of causing LV chamber obliteration during systole. So far, there are reports presented with isolated papillary muscle hypertrophy and normal LV wall thickness. Herein, we report a case of morphological papillary muscle anomalies featured by dynamic LV mid-wall obstruction, with subjective exertional chest pain and dyspnea. We further observed in our case that severely impaired LV diastolic function and clinical heart failure [14] may occur as a consequence of excessive, redundant papillary muscle mass with normal LV mass [7].

Current guidelines for LV mass calculation do not take papillary muscle mass into account, while our relevant findings may suggest include papillary muscles in LV mass assessment, especially for these morphologically abnormal papillary muscle subjects.

Solitary papillary muscle hypertrophy as an uncommon variant form of HCM with coexisted additional, accessory papillary muscle may develop abnormally high resting LV mid-wall pressure gradient without SAM or significant regional LV wall hypertrophy. Cardiac MRI and RT-3DE may have their peculiar roles in uncovering these cardiac morphological/structural anomalies aiming for a more comprehensive analysis on their spatial relationships.

In conclusion, we recommended that for subjects with unusually high LV mid-wall or LVOT pressure gradient with unrevealing regional LV segment anomalies, RT-3DE and MRI should be considered as a screening tool to disclose these under-diagnosed, specific HCM subjects in daily routine.

## Consent

Written informed consent was obtained from the patient for publication of this Case report and any accompanying images. A copy of the written consent is available for review by the Editor-in-Chief of this journal.

## Abbreviations

LV: Left ventricle; RT: Real time; 3DE: three-dimensional echocardiography; MRI: magnetic resonance imaging; PM: Papillary muscle; HCM: Hypertrophic cardiomyopathy; HOCM: Hypertrophic obstructive cardiomyopathy; LVOT: Left ventricular outflow tract; SAM: Systolic anterior motion; DHE: Delayed hyperenhancement.

## Competing interests

The authors declare that they have no competing interests.

## Authors’ contributions

KTS was the primary author of the text. CHY assisted in image acquisition and retouching. CLH conceived of the report, provided the images, and modified the manuscript. JYH provided additional supervision. All authors have read and approved the final manuscript.

## Pre-publication history

The pre-publication history for this paper can be accessed here:

http://www.biomedcentral.com/1471-2261/14/34/prepub

## Supplementary Material

Additional file 1 Real time 3D Echocardiography, four chamber view. Click here for file

Additional file 2 Real time 3D Echocardiography, short axis view. Click here for file

Additional file 3 Real time 3D echocardiography sequences in short axis view, divided the left ventricle into 9 slices during end-diastolic phase. Click here for file
