# Supplementary figures and images for: Solitary accessory and papillary muscle hypertrophy manifested as dynamic mid-wall obstruction and symptomatic heart failure: diagnostic feasibility by multi-modality imaging
Source: BMC Cardiovasc Disord. 2014 Mar 10;14:34. doi: 10.1186/1471-2261-14-34 (PMC4015509; doi:10.1186/1471-2261-14-34)

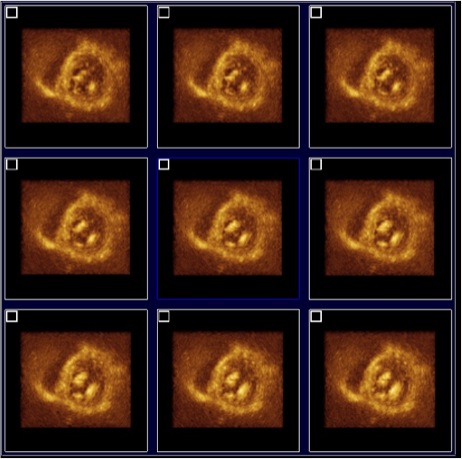

Supplement: Additional file 3 — Real time 3D echocardiography sequences in short axis view, divided the left ventricle into 9 slices during end-diastolic phase. [file 1471-2261-14-34-S3.jpeg]
